# Two is company: The posterior cerebellum and sequencing for pairs versus individuals during social preference prediction

**DOI:** 10.3758/s13415-023-01127-y

**Published:** 2023-10-11

**Authors:** Naem Haihambo, Qianying Ma, Kris Baetens, Tom Bylemans, Elien Heleven, Chris Baeken, Natacha Deroost, Frank Van Overwalle

**Affiliations:** 1 Department of Psychology and Center for Neuroscience, Vrije Universiteit Brussel, Pleinlaan 2, B-1050 Brussels, Belgium; 2 Department of Psychiatry, University Hospital UZBrussel, Brussels, Belgium; 3 Department of Electrical Engineering, Eindhoven University of Technology, Eindhoven, Netherlands

**Keywords:** Social interactions, Posterior cerebellum, Social action prediction, Preferences, Social action sequencing

## Abstract

Previous studies have identified that the posterior cerebellum, which plays a role in processing temporal sequences in social events, is consistently and robustly activated when we predict future action sequences based on personality traits (Haihambo Haihambo et al. *Social Cognitive and Affective Neuroscience 17*(2), 241–251, 2022) and intentions (Haihambo et al. *Cognitive, Affective, and Behavioral Neuroscience 23*(2), 323–339, 2023). In the current study, we investigated whether these cerebellar areas are selectively activated when we predict the sequences of (inter)actions based on protagonists’ preferences. For the first time, we also compared predictions based on person-to-person interactions or single person activities. Participants were instructed to predict actions of one single or two interactive protagonists by selecting them and putting them in the correct chronological order after being informed about one of the protagonists’ preferences. These conditions were contrasted against nonsocial (involving objects) and nonsequencing (prediction without generating a sequence) control conditions. Results showed that the posterior cerebellar Crus 1, Crus 2, and lobule IX, alongside the temporoparietal junction and dorsal medial prefrontal cortex were more robustly activated when predicting sequences of behavior of two interactive protagonists, compared to one single protagonist and nonsocial objects. Sequence predictions based on one single protagonist recruited lobule IX activation in the cerebellum and more ventral areas of the medial prefrontal cortex compared to a nonsocial object. These cerebellar activations were not found when making predictions without sequences. Together, these findings suggest that cerebellar mentalizing areas are involved in social mentalizing processes which require temporal sequencing, especially when they involve social interactions, rather than behaviors of single persons.

## Introduction

Preferences—we have them, and others do too. In order to have smooth and efficient social interactions, we rely on our assumptions of what others are thinking, including what they prefer. The process of inferring others’ mental states, such as desires, traits, goals, and preferences, is termed mentalizing. This process makes it possible for us to anticipate what happens next, thereby predicting future social interactions (Bubic et al., 2010; Frith & Frith, 2006b; Molinari & Masciullo, 2019; Seif et al., 2021; Siciliano et al., 2023), which is ultimately the goal of mentalizing (Frith & Frith, 2006a).

Mentalizing is supported by a group of brain areas in the cerebral cortex, such as the medial prefrontal cortex (mPFC), temporoparietal junction (TPJ), and precuneus, collectively referred to as the mentalizing network (Molenberghs et al., 2016; Schurz et al., 2014; Van Overwalle, 2009). One of the key areas in the mentalizing network is the TPJ, which is thought to play a crucial role in mentalizing by integrating social and perceptual information to generate inferences about other peoples' mental states (Saxe & Kanwisher, 2003). The mPFC seems to play a role in making inferences about others’ stable characteristics, such as personality traits (Amodio & Frith, 2006) and preferences (Izuma & Adolphs, 2013; Kang et al., 2013; Tamir & Mitchell, 2010; Tusche et al., 2013; Vijayakumar et al., 2021). Of note, the mentalizing network largely overlaps with the default mode network (Andrews-Hanna et al., 2014; Fox et al., 2015; Yeo et al., 2011), which is activated at wakeful rest when we mind-wander, think about the past, and daydream about the future (Buckner et al., 2008).

In recent years, there has been a growing interest in the role of the cerebellum in mentalizing (Sokolov, 2018; Van Overwalle et al., 2014; Van Overwalle, D’aes et al., 2015b; Van Overwalle, Ma et al., 2020), after a number of large-scale studies provided strong evidence for a role that goes beyond motor, and even cognitive or affective processes (Buckner et al., 2011; Van Overwalle et al., 2014). It has been hypothesized that the posterior cerebellum plays a crucial role in identifying sequences (i.e., temporal order) of social information and encoding this information into internal models (*sequence detection hypothesis;* Van Overwalle, Manto et al., 2019), which are then automatized after repeated exposure, and used to identify and anticipate future social (inter)actions (Gatti et al., 2021; Leggio & Molinari, 2015; Van Overwalle, De Coninck et al., 2019a; Van Overwalle, Manto et al., 2020). Suppose you are having a conversation with a friend in a noisy coffee shop. Your cerebellar internal models help you to predict what your friend is going to say next based on the words and phrases they have used so far, even if you cannot hear them perfectly. This prediction allows you to anticipate their response and adjust your own behavior accordingly, such as by nodding, responding, or asking a clarifying question. Importantly, the posterior cerebellum Crus 1 and 2 have consistently been activated in a variety of studies involving action sequences that require social mentalizing as opposed to when these actions do not require attention to sequences or involve nonsocial events (e.g., objects; for an overview, Van Overwalle et al., 2021). In particular, recent studies have demonstrated that mentalizing about social beliefs (Heleven et al., 2021; Ma et al., 2021), goals (Li et al., 2021, 2022), and personality traits activates the cerebellar Crus when sequencing is a crucial aspect of the task compared with nonsequencing and nonsocial control conditions. These findings were further supported by a meta-analysis which found that the cerebellar Crus 2 was preferentially recruited in mentalizing related tasks (Van Overwalle, Ma, & Heleven, 2020a). Of note, connectivity studies have shown that activation of the Crus area during identification of action sequences runs in synchrony with cerebral mentalizing areas, such as the TPJ (Ma et al., 2023), precuneus and mPFC (Pu, Ma, Haihambo et al., 2022) via bidirectional closed loops between these cerebellar and cerebral areas. This further highlights that cerebral and cerebellar mentalizing areas act together to process social information that requires social mentalizing.

Important for the current study is that the cerebellar Crus also was activated when participants predicted future social actions based on previous information about agents’ traits (Haihambo et al., 2021). In addition, the cerebellar lobule IX in the inferior posterior cerebellum has been found to be activated when predictions were based on others’ traits and intentions (Haihambo et al., 2022, 2023). This is in line with earlier research demonstrating activation of lobule IX during future oriented thinking and prediction of social processes (Addis et al., 2009).

Although there is accumulating evidence demonstrating that cerebral and cerebellar mentalizing areas are involved in predicting social interactive sequences (Haihambo et al., 2021, 2023), a limitation is that they always involved the interaction between two agents and do not include a noninteractive social condition (i.e., mentalizing about a single person). Other studies (Baetens et al., 2013) have found cerebellar mentalizing areas to be involved in mentalizing about a single person; however, these studies do not make a direct comparison to interactive scenarios. For a more comprehensive understanding of the role of the cerebellum in social prediction, it is crucial to disentangle observations of social interactions from single person observations.

A meta-analysis by Arioli and Canessa (2019) found that observing social interactions was supported by the cerebral *mentalizing* network and a so-called *social interaction* network, which included key nodes of the action observation (or mirror) network (see also meta-analyses by Molenberghs et al., 2012; Van Overwalle & Baetens, 2009). Although this meta-analysis demonstrated the involvement of the action observation network, many of the studies included visuospatial stimuli (e.g., static images or dynamic videos) and visual observations of past or present social interactions, so that the perception of biological movement alone may have increased the involvement of the action observation network (Van Overwalle & Baetens, 2009). Additionally, the studies included in this analysis did not explicitly require participants to actively identify, memorize, or produce temporal sequences of events. Consequently, we still know little about the neural correlates of predicting the temporal order of social interactions.

## Present study

In a previously mentioned study, Haihambo et al. (2022) investigated the neural correlates involved in predicting future social action sequences based on a variety of stable trait information about another person. Both cerebral (mPFC, TPJ, and precuneus) and cerebellar (Crus 1, 2, and lobule IX) mentalizing regions were involved. In a follow-up study, Haihambo et al. (2023) investigated predictions based on temporal goal-directed social intentions (i.e., to be honest or deceitful). The same activations were found, except that only lobule IX in the posterior cerebellum was activated and no Crus 1 or 2 activations were observed. Importantly, in line with the sequencing detection hypothesis of the cerebellum, cerebellar mentalizing areas were more activated in a sequencing versus a nonsequencing condition, whereas cerebral mentalizing areas were activated in social (versus nonsocial) conditions irrespective of sequencing conditions (Haihambo et al., 2022, 2023).

In the current study, we investigated cerebellar involvement in social action prediction. Specifically, we investigated cerebellar involvement in social action sequencing based on others’ known preferences. Additionally, we sought to investigate for the first time whether the cerebellum is sensitive to descriptions of interactions between two persons or the sole action of a single individual. To do this, we used a similar paradigm as in our previous prediction studies (Haihambo et al., 2022, 2023). Specifically, we presented participants with a prompt sentence describing protagonists and their preference (e.g., Ytol prefers hiking), followed by randomly presented sentences describing protagonists’ engaging in an activity relevant to this preference alone (Solo) or with another agent (Interactive). We also included distractor sentences describing activities related to an alternative preference. Participants had to predict protagonists’ behaviors based on their preferences by selecting the preference-consistent sentences and put these in the correct temporal order. To verify selective involvement in social mentalizing and sequencing in social action prediction, we included a non-social control condition (including objects) and a nonsequencing control condition (selecting the correct preference-related behaviors without generating a sequence).

We put forward the following hypotheses. First, our novel hypothesis is that making predictions about future social actions involving two interacting protagonists (Interactive) will generate more mentalizing activity than making these predictions involving a solo protagonist (Solo). This is because observing and predicting interactions requires tracking two, instead of a single, mental states. Specifically, we expect an increase in activation in posterior cerebellar Crus areas (Lewis et al., 2017) and also of cerebral mentalizing areas, including the TPJ which is responsible for understanding mental beliefs of others and tends to increase activation when more persons are involved (Özdem et al., 2019) and more dorsal areas in the mPFC associated with making inferences about personality traits (see meta-analysis by Van Overwalle, 2009). Second, in line with recent findings of the cerebellum in social processing (Van Overwalle et al., 2014; Van Overwalle, Baetens, et al., 2015a; Van Overwalle, D’aes, & Mariën, 2015b), our general hypothesis is that mentalizing areas in the cerebellum, specifically, the posterior cerebellar Crus 1 and Crus 2 and inferior posterior lobule IX, will be preferentially selectively recruited in predicting the preferences of social agents when sequences are required, compared to predicting the outcome of nonsocial or nonsequencing events. We expect that, irrespective of sequencing, predicting social events will also invite activation of cerebral mentalizing areas in the TPJ, mPFC, and precuneus, as well as in the cerebellum.

## Method

### Participants

Participants in this study were 27 healthy, right-handed, Dutch-speaking volunteers (13 males; mean age 21 years, standard deviation [SD] = 1.8 years). This number excludes two participants whose data was corrupted. The sample size was determined based on earlier work on preference mentalizing (e.g., Iacoboni et al., 2004, N = 13; Izuma & Adolphs, 2013, N = 20; Kang et al., 2013, N = 22), social sequencing (e.g., Heleven et al., 2019, N = 28) mentalizing for two persons (Özdem et al., 2019, N = 28), and studies using a similar paradigm (Haihambo et al., 2022, N = 27, 2023, N = 26). All participants had normal or corrected-to-normal vision and reported no neurological or psychiatric disorders. Informed consent was obtained following the guidelines of the Medical Ethics Committee at the Ghent University Hospital, where the study was conducted. Participants were given 20 euros and reimbursed for transportation costs in exchange for their participation.

### Stimulus material

**Fig. 1:**
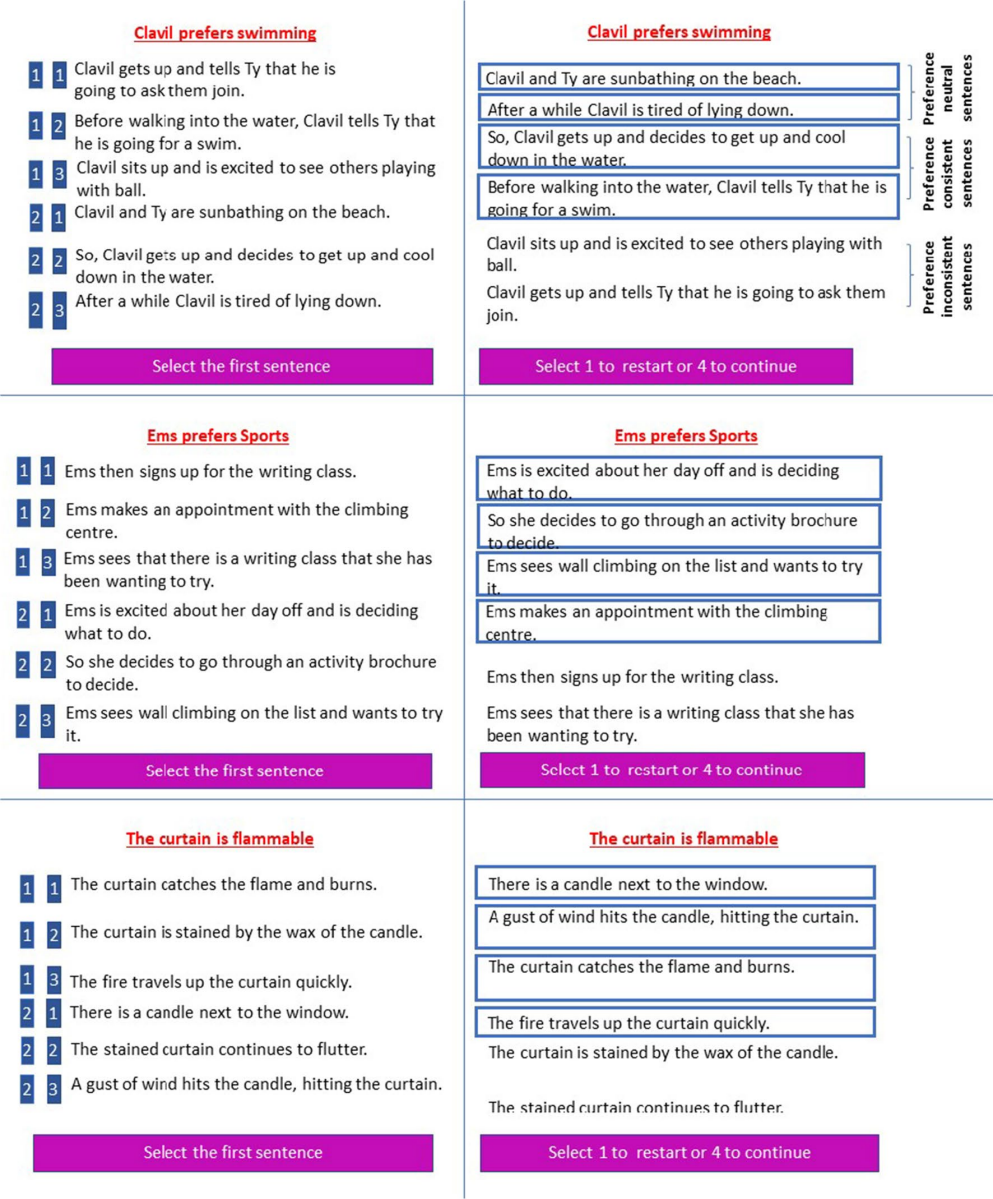
Example of a trial from the Sequencing condition. Interactive (top panel), Solo (middle panel) and Nonsocial (bottom panel) conditions. In red at the top are the prompt sentences. **Left:** Participants were presented six action sentences (randomly ordered) and were required to select the four sentences that fit best with the person’s preference/object feature, and to order them in the correct order (ignoring the inconsistent sentences) using two consecutive button presses on a four-button response box (with responses indicated on a blue background on the left of the screen). **Right:** The correct ordering as chosen by a participant (the four sentences were ordered from top to bottom in the order of selection and marked by squares surrounding them)

The present study used an adjusted social action prediction paradigm from previous studies by Haihambo et al. (2022, 2023). In the present task, participants were presented with Interactive, Solo (noninteractive) and nonsocial sentence sets. The Interactive and Solo sentences consisted of a prompt sentence and six preference-implying sentences. In the prompt sentence, participants were presented with a protagonist and their preference (e.g., Ytol prefers hiking). The prompt sentence was followed by six behavioral sentences regarding this protagonist that described interactions with another protagonists (Interactive) or involved only this single protagonist (Solo), in relation to the preference described in the prompt sentence (Fig. 1). The names used in the sentences were fictional names to eliminate potential confounds of biases related to known persons. These six sentences were made up of two neutral, two preference-consistent, and two preference-inconsistent sentences. The two inconsistent sentences included the same number of protagonists as in the consistent sentences, although they were not related to the preference of the protagonist(s) stated earlier, and hence, these sentences served as distractors. The neutral sentences were logically part of the story but were unrelated to any preference.

The non-social control sentence sets consisted of a prompt sentence involving an object, followed by six sentences regarding the object. Specifically, in the prompt sentence, participants were presented with an object and its characteristic (e.g., the curtain is flammable). Similar to the social counterpart, this prompt sentence was followed by six non-social sentences that were made up of two neutral, two consistent, and two inconsistent sentences relative to the object characteristic presented in the prompt sentence (Fig. 1).

**Fig. 2:**
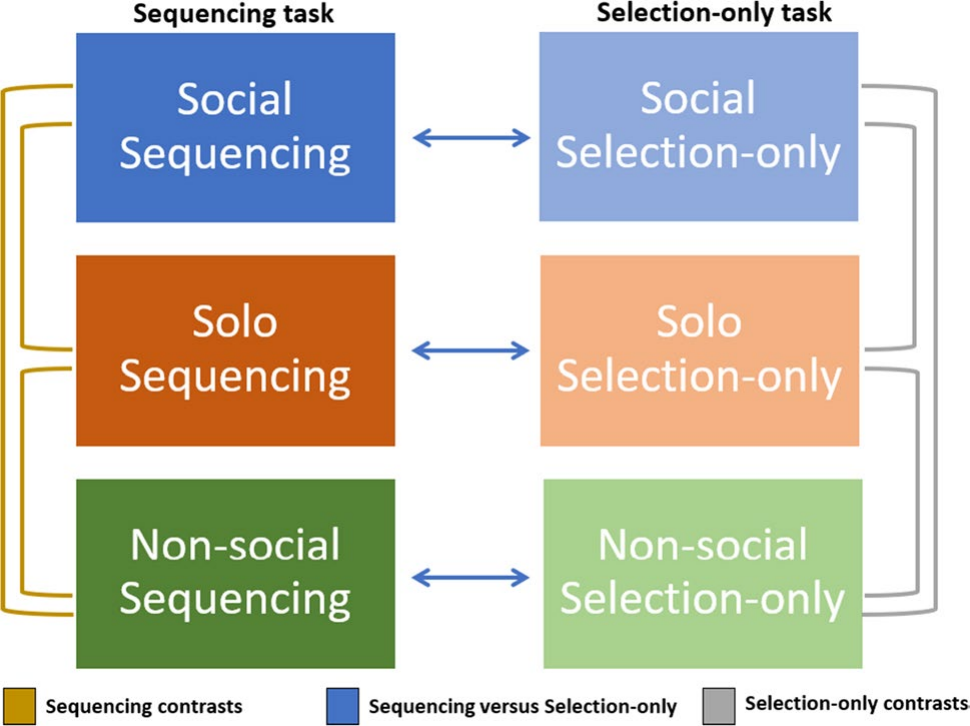
Illustration of the tasks, conditions, and contrasts in this study. **Left:** Sequencing task where participants are required to order the Social (top), solo (middle), and nonsocial sentences(bottom) based on the person’s preference or object’s feature. **Right:** Selection-only task where participants are required to choose the correct preference or object feature from two options. The legend at the bottom refers to the arrows: Arrows in gold represent contrasts within the sequencing conditions, blue arrows indicate contrasts between the sequencing and selection-only conditions, and grey arrows represent contrasts within the selectin-only condition

All Interactive, Solo and Nonsocial sentences were randomly distributed between two tasks: a Sequencing task and a Selection-only (i.e., non-sequencing control) task. This resulted in a Domain (Interactive, Solo and Nonsocial) and Task (Sequencing and Selection-only) design consisting of six conditions, illustrated in Fig. 2. The basic sentence structures were identical between conditions, with the exception that the social conditions included one or two social agents performing social actions, whereas the nonsocial conditions included objects in relation to their environment. All sentences were newly developed for this study. All social and nonsocial sentences were pilot tested for sequencing accuracy.

In a pilot study, 78 participants (27 males; mean age 19 years, SD = 1.1 years) were presented with the six action sentences and were instructed to select the four preference consistent sentences in the correct chronological order, leaving the two inconsistent sentences out. Participants also were asked how consistent a sentence was to the preference on a 7-item scale with anchors: 1 = inconsistent; 4 = neutral; 7 = consistent. Sentence sets were included if the correct sequence was identified at least 65% of the time and received a mean consistency score of 3.5-4.5 for neutral sentences, <3.5 for inconsistent sentences, and >5.5 for consistent sentences. The sequencing accuracy score of minimally 65% allowed for sufficient variation in difficulty. Participants in this pilot study did not participate in the fMRI study.

### Procedure

The procedure is identical to the procedures used in our previous studies involving trait prediction (Haihambo et al., 2022) and intention prediction (Haihambo et al., 2023). Participants were informed that the experiment included two tasks: Sequencing and Selection-only. In the Sequencing task, participants were instructed to “choose the four sentences that fit the preference of the person or characteristic of the object and put them in the correct chronological order.” In the Selection-only task, participants were told that “the sentences are already put in the correct order and that they only had to “select the four sentences that fit the preference of the person or characteristic of the object.” In both tasks, participants were further told to “execute this task as accurately and as quickly as possible. Your time is measured from the presentation of the event until you indicate that you are ready.” To avoid spill-over learning effects, the Selection-only task always came after the Sequencing task, so that participants were not primed with the correct structure of already ordered sentences in the Selection-only task. The Interactive, Solo, and Non-social sentences were presented in a random order for each participant within each task.

In each trial of the Sequencing task, participants were first shown a preference of a protagonist (Interactive and Solo conditions) or a characteristic of an object (Nonsocial condition; Fig. 1). This prompt sentence appeared in red on the top of the screen, where it remained for the entire duration of the trial. After 1,000 ms, the first of six sentences was shown on the screen, followed by the remaining five sentences which appeared one-by-one after 1,300 ms each, based on previous prediction studies (Haihambo et al., 2022, 2023), because the procedure and sentence length were comparable. The six sentences were presented in random order for each participant and for each trial. After individual presentation, all sentences were shown together on screen in the same random order along with numbers on the side of the sentences used for responding (Fig. 1). Then, a prompt to select the first sentence appeared at the bottom of the screen, followed by a prompt to select the next sentence, until they selected all sentences. No duration was set for completing this task. Once four sentences were selected, participants were then prompted to select “1 to restart or 4 to continue.” At the end of each trial, a confidence question appeared: “how confident are you about your answer” and a 4-point rating scale ranging from 1 = not at all to 4 = very much. Participants responded with a button press using an MRI compatible four button response box positioned in their left hand. All trials and confidence ratings were preceded by a blank screen with a fixation cross, jittered randomly between 1–2 s. The same procedure was used for the Interactive, Solo, and Nonsocial conditions.

In the Selection-only task, the procedure was identical to the Sequencing task, with the exception that participants did not have to put the sentences into the correct chronological order and individual sentences were presented one-by-one for 1,100 ms (as in the previous trait prediction study) instead of 1,300 ms used for sequencing, because participants did not need to order the sentences, hence requiring less time. Specifically, participants were presented with two neutral sentences in their correct chronological order, followed by a pair of consistent or inconsistent sentences each in their correct chronological order. Participants had to select only the set of preference/characteristic consistent sentences by selecting “1” or “2,” followed by a confidence rating as in the Sequencing task. The entire experiment lasted approximately 45 minutes.

Before entering the scanner, participants were presented with a short practice version of the experiment to practice the response presses and order the sentences. They were presented with two Sequencing and two Selection-only trials that were not part of the fMRI experiment, followed by confidence ratings. The whole experiment outside and inside the scanner was presented in E-Prime 3.0 (www.pstnet.com/eprime; Psychology Software Tools), running on a Windows 10 computer.

In total, participants completed 60 trials, each consisting of six different sentences. Each Sequencing or Selection-only task consisted of ten Interactive, ten Solo, and ten Nonsocial trials.

### Imaging procedure and pre-processing

Images were collected with a Siemens Magnetom Prisma fit scanner system (Siemens Medical Systems, Erlangen, Germany) using a 64-channel radiofrequency head coil. Stimuli were projected onto a screen at the end of the magnet bore, which participants viewed by way of a mirror mounted on the head coil. Participants were placed headfirst and supine in the scanner bore and were instructed not to move their heads to avoid motion artifacts. Foam cushions were placed within the head coil to minimize head movements. First, high-resolution anatomical images were acquired using a T1-weighted 3D MPRAGE sequence [repetition time (TR) = 2,250 ms, echo time (TE) = 4.18 ms, inversion time (TI) = 900 ms, field of view (FOV) = 256 mm, flip angle = 9°, voxel size = 1 × 1 × 1 mm]. Second, a fieldmap was calculated to correct for inhomogeneities in the magnetic field (Cusack and Papadakis, 2002). Third, whole brain functional images were collected in a single run by using a T2*-weighted gradient echo sequence, sensitive to blood oxygen level-dependent (BOLD) contrast (TR = 1,000 ms, TE = 31.0 ms, FOV = 210 mm, flip angle = 52°, slice thickness = 2.5 mm, distance factor = 0%, voxel size = 2.5 × 2.5 × 2.5 mm, 56 axial slices, acceleration factor GeneRalized Autocalibrating Partial Parallel Acquisition (GRAPPA) = 4).

SPM12 (Wellcome Department of Cognitive Neurology, London, UK) was used to process and analyze the fMRI data. To remove sources of noise and artifact, data were preprocessed. Functional data was corrected for differences in acquisition time between slices for each whole-brain volume, realigned to correct for head movement, and co-registered with each participant’s anatomical data. Then, the functional data was transformed into a standard anatomical space (2-mm isotropic voxels) based on the ICBM152 brain template (Montreal Neurological Institute). Normalized data were then spatially smoothed (6-mm full width at half-maximum, FWHM) using a Gaussian Kernel. Finally, using the Artifact Detection Tool (ART; http://web.mit.edu/swg/art/art.pdf;http://www.nitrc.org/projects/artifact_detect), the data was examined for excessive motion artifacts and for correlations between motion and experimental design, and between global mean signal and experimental design. Outliers were identified in the temporal differences series by assessing between-scan differences (Z-threshold: 3.0 mm, scan-to-scan movement threshold: 0.5 mm; rotation threshold: 0.02 radians). These outliers were “omitted” from the analysis by including a single regressor for each outlier. A default high-pass filter was used of 128 s, and serial correlations were accounted for by the default auto-regressive (AR) model.

### Statistical analysis of behavioral data

Accuracy for Sequencing tasks was calculated by giving 1 point for each selected sentence that matched the prompt and in the correct order, and 0 points for an incorrect response, with a maximum of 4 points. For the Selection-only tasks was calculated by giving 1 point for selecting the sentence set that matched the prompt and 0 for an incorrect response, with a maximum of 1 point. The response time (RT) was calculated by timing the whole trial; i.e., starting after all six sentences were presented on screen for the first time and the prompt to select or sequence the sentences appeared, until the selection of the final (fourth) sentence before pressing “4 to continue.”

A 3 x 2 repeated measure analysis of variance (ANOVA) with Domain (Interactive, Solo, and Nonsocial) and Task (Sequencing vs. Selection-only) as within-participants factors was conducted on accuracy and RT using IBM SPSS Statistics 27 software. The alpha level for pairwise comparisons was set at 0.05 and is reported when significant interactions are revealed.

#### Statistical analysis of neuroimaging data

At the first (single participant) level, for each task, the event-related design was modelled for each of the six conditions (i.e., Interactive, Solo and Non-social Sequencing; Interactive, Solo, and Nonsocial Selection-only). The onset of each trial was set after all six sentences were presented together on screen and the prompt to select or sequence the sentences appeared. The presentation of each sentence was relatively short, so that little time was left for anything else other than reading. Hence, although participants could start eliminating inconsistent sentences as soon as they saw one sentence, properly sequencing the sentences was only possible after all sentences were carefully read. Based on considerations of how response processes might have evolved during a trial and our aim to select equivalent timings for fMRI analysis across conditions, duration was set from the onset of the trial (i.e., after the prompt sentence and all six sentences were presented) until the time participants made their final selection (i.e., selection of four sentences reflecting the preference in the Sequencing and the Selection-only tasks) and pressed the “continue” button. This timing reflects the same process across the two tasks. All trials were analyzed, irrespective of whether selection or sequencing was correct, because we assumed that participants’ selection and sequencing was based on what they believed to be correct. When a trial was canceled and redone, analysis was performed on the responses and timing of the final sentence selection. The occurrence of participants redoing a trial was quite low, with participants cancelling an average of 1.9 trials for the Interactive Sequencing conditions, 2 trials in the Solo Sequencing condition and 1.7 trials in the Nonsocial Sequencing condition. The occurrence of redoing trials in the Selection-only tasks was 0 across all conditions.

At the second (group) level, a whole-brain random effects analysis using one-way within-participants ANOVA. Significance was set at the cluster-defining uncorrected threshold of *p* < 0.001, followed by a cluster-wise FWE corrected threshold of *p* < 0.05 with a minimum cluster extent of 10 voxels. We also tested our hypotheses more directly by performing a Region of Interest (ROI) analysis, using spheres of 8 mm centered on *a priori* MNI coordinates for the cerebellar Crus 1 and 2 (±40 −70 −40 and ±24 −76 −40, respectively; Van Overwalle, Ma et al., 2020). For lobule IX, we used the average peak coordinates from our previous activation studies (0 −52 −40; Haihambo et al., 2022, 2023). Additionally, we included cerebral mentalizing area coordinates for the mPFC (0 50 20), dmPFC (0 50 35), vmPFC (0 50 5), TPJ (±50 −55 25), and precuneus (0 −60 10) from meta-analyses on social cognition (Van Overwalle, 2009; Van Overwalle & Baetens, 2009). These coordinates are also listed in Table 1. ROI analyses were done using a small volume (rather than whole-brain volume) correction for multiple comparisons with the same thresholds as we did the whole-brain analysis.

**Table 1:** Regions of interest

Brain region	ROI coordinates		
	x	y	z
Cerebellar mentalizing areas			
Crus 1	±40	−70	−40
Crus 2	±25	−75	−40
Lobule IX	0	−52	−44
Cerebral mentalizing areas			
dmPFC	0	50	35
mPFC	0	50	20
vmPFC	0	50	5
TPJ	±50	−55	25
Precuneus	0	−60	10

Regions of interest for cerebellar (Haihambo et al., 2022; Haihambo et al., 2023; Van Overwalle, Ma, & Heleven, 2020a) and cerebral (Van Overwalle, 2009; Van Overwalle & Baetens, 2009) mentalizing areas identified from previous meta-analysis and activation studies

## Results

### Behavioral results

Although we do not have a specific hypothesis on behavioral outcomes, we report these results to be exhaustive. A 3 x 2 repeated ANOVA with Domain (Interactive vs. Solo vs. Nonsocial) and Task (Sequencing vs. Selection-only) within-participants factors was conducted on accuracy and reaction times (RT). An overview of the means and standard deviations are listed in Table 2.

**Table 2:** Means and standard deviations of accuracy and reaction times for the Preference Prediction task

Task	Domain	Accuracy		Response time	
		Mean	SD	Mean	SD
Sequencing	Interactive	3.79	0.25	38.99	11.32
	Solo	3.76	0.27	41.64	9.88
	Nonsocial	3.59	0.31	33.36	12.96
Selection-only	Interactive	0.99	0.03	2.98	1.92
	Solo	0.96	0.09	4.63	2.67
	Nonsocial	0.94	0.07	2.87	3.32

For the Sequencing tasks, accuracy was scored with 1 point allocated for each correctly selected and sequenced sentence, with a maximum score of 4. For the Selection-only task, accuracy was scored 1 for a correct sentences and 0 for an incorrect response. Reaction time was measured in seconds. SD = standard deviation

***Accuracy***. For accuracy, results showed significant main effects for Domain [F(2, 56) = 13.40, *p* < 0.001, ηp^2^ = 0.32] and Task [F(1, 28) = 6.63, *p* < 0.05, ηp^2^ = 0.19], indicating that accuracy differed significantly across domains with participants performing better in the Solo conditions (Sequencing: M = 94%, SD = 7%; Selection-only: M = 96% SD = 9%), followed by the Nonsocial conditions (Sequencing: M = 98%, SD = 4%; Selection-only: M = 93% SD = 7%), and least accurate in the Interactive conditions (Sequencing: M = 95%, SD = 6%; Selection-only: M = 90% SD = 8%). We did not find a significant interaction between Domain and Task, however (*p* > 0.05).

***RT.*** For RT, results showed significant main effects for Domain [F(2, 56) = 38.86, *p* < 0.001, ηp2 = 0.59] and Task [F(1, 28) = 336.92, p < 0.001, ηp2 = 0.92]. This was further qualified by further post hoc pairwise comparisons with a Bonferroni correction, which revealed that participants were slower in the sequencing and faster in the selection-only tasks (*p* < 0.001), which was expected due to task requirements. In both tasks, there was a significant interaction between Domain and Task [F(2, 56) = 29.67, *p* < 0.001, ηp^2^ = 0.51]. Specifically across both tasks, participants were fastest in the in the Solo conditions (Sequencing: M = 33s SD = 10s; Selection-only: M = 3s SD = 3s), followed by the Interactive conditions (Sequencing: M = 38s SD = 12s; Selection-only: M = 3s SD = 2s), and were slowest in the Nonsocial condition (Sequencing: M = 42s SD = 13s; Selection-only: M = 5s SD = 3s).

Based on G*Power analysis (3.1.9.4; Faul et al., 2007), and considering our sample size of 27, an effect size (Cohen's d) of 0.25, and an alpha level of 0.05, the post-hoc power analysis revealed a statistical power of 0.80, indicating an 80% chance of detecting a true effect, given the parameters of our study.

### fMRI results

To investigate the social and sequencing functions of the cerebellum, we computed a number of contrasts comparing Interactive and Solo versus Nonsocial conditions, Interactive versus Solo conditions, and Sequencing versus Selection-only conditions, while holding all other manipulations constant. We performed whole brain and ROI analysis on all contrasts and also report the reverse contrasts to exhaustively test that the hypothesized effects are found only in the expected direction of the comparison. For ease of presentation, we describe all peak and subpeak activations in the cerebellum and mentalizing areas of interest, and only peak activations in other areas. We report the results of the Social (Interactive and Solo) versus Nonsocial, Interactive versus Solo, and Sequencing versus Selection-only contrasts in this order.

### Replication of previous findings: Social (Interactive and Solo) versus Non-social contrasts

Our general hypothesis was that there would be activations in the posterior (Crus 1 and 2) and inferior posterior cerebellum (lobule IX) in all Social > Nonsocial contrasts during Sequencing (but not during Selection-only), while both cerebellar and cerebral mentalizing areas (TPJ, mPFC, and precuneus) would be revealed in all Social > Nonsocial contrasts, irrespective of sequencing. To test this hypothesis, we first evaluate the effects of Interactive and Solo conditions in comparison with their Nonsocial counterparts for the Sequencing conditions, and then for the Selection-only conditions.

#### Interactive Sequencing versus Nonsocial Sequencing

**Fig. 3:**
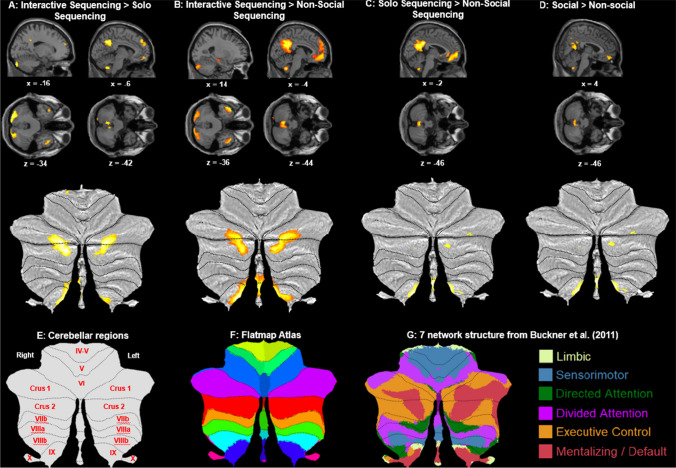
Sagittal and transverse views of the experimental contrasts visualized at a whole-brain FWE corrected threshold of *p* < 0.05, together with visualization on SUIT flatmaps of the cerebellum. **A:** Interactive Sequencing > Solo Sequencing contrast showing Crus 1, Crus 2, and lobule IX activation. **B:** Interactive Sequencing > Nonsocial Sequencing contrast showing Crus 1, Crus 2, and lobule IX activation. **C:** Interactive Sequencing > Nonsocial Sequencing contrast, showing lobule IX activation. **D:** Social > Nonsocial main effects contrast, showing cerebellar lobule IX activation. **E:** Flatmap showing all regions of the cerebellum **F:** SUIT flatmap atlas showing cerebellar lobes identified by color **G:** the 7-network structure from Buckner et al. (2011) shown on a cerebellar flatmap (http:// www. diedrichsenlab.org/imaging/Atlas Viewer/viewer. html) along with the color legend denoting the functional network each color represents

The whole brain analysis of the Interactive Sequencing > Nonsocial sequencing contrast (Fig. 3B; Table 3) revealed, as predicted, activations in cerebellar mentalizing areas in the left Crus 1, bilateral Crus 2, and cerebellar lobule IX. Additionally, ROI analysis revealed activations in the mPFC, dorsal mPFC (dmPFC), and ventral mPFC (vmPFC), as expected. We also found activations in hypothesized cerebral mentalizing areas in the bilateral TPJ and precuneus. We found further activations in the bilateral angular gyrus, bilateral posterior middle temporal gyrus (aMTG), and mid orbital gyrus. The opposite contrast (Interactive Sequencing < Nonsocial Sequencing) revealed activations in the left anterior middle temporal gyrus (pMTG), left supramarginal gyrus, and left inferior frontal gyrus (IFG) pars triangularis.

**Table 3:** Whole brain and ROI analyses comparing Sequencing versus Selection-only conditions

Brain label	MNI coordinates			Voxels	Max t
	x	y	z		
Interactive Sequencing > Nonsocial Sequencing					
R Cerebellum Crus 2	14	−90	−36	334	7.46***
R Cerebellum Crus 1	28	−82	−30		6.41***
L Cerebellum Crus 2	−16	−86	−38	350	6.34***
L Cerebellum Crus 2	−24	−84	−34		6.04***
L Cerebellum Crus 2	−34	−80	−34		5.62***
R Angular Gyrus, including TPJ	54	−64	28	413	6.63***
L Angular gyrus, including TPJ	−38	−58	26	941	7.13***
L Angular gyrus	−42	−64	38		6.76***
L Angular gyrus	−50	-68	36		5.46***
L cerebellum IX	−4	−54	-44	307	8.05***
R cerebellum IX	8	−50	-42		5.89***
R PCC	2	−48	30	2984	9.64***
L precuneus	−8	−58	36		9.22***
L MCC	−10	-48	32		8.96***
L middle temporal gyrus	−62	-8	-14	1778	10.68***
L middle temporal gyrus	−58	-2	-22		9.89***
L medial temporal pole	−48	16	−30		9.38***
R middle temporal gyrus	58	-6	−20	1,363	8.82***
R medial temporal pole	48	16	−30		8.28***
R middle temporal gyrus	58	2	−22		7.96***
ROI: mPFC	6	52	22	274	5.25**
ROI: dmPFC	−6	58	36	270	5.36**
ROI: vmPFC	0	56	−2	195	6.08***
L mid orbital gyrus, including vmPFC	0	60	−6	2,971	8.06***
R rectal gyrus	2	48	−18		7.68***
L rectal gyrus	0	40	−16		7.22***
Interactive Sequencing < Nonsocial Sequencing					
L middle temporal gyrus	−56	−60	0	485	7.47***
L middle temporal gyrus	−46	−56	4		5.53**
L supramarginal Gyrus	−60	−38	36	730	7.17***
L supramarginal Gyrus	−62	−26	32		5.60**
L inferior parietal lobule	−60	−32	42		5.44**
L IFG p. triangularis	−44	36	18	550	6.75***
L IFG p. triangularis	−48	40	6		5.86***
L IFG p. triangularis	−38	38	8		5.13**
Solo Sequencing > Nonsocial Sequencing					
L cerebellum IX	−2	−56	−46	100	5.20**
L precuneus	−2	−52	20	1595	7.20***
L PCC	−2	−42	32		7.00***
L precuneus	0	−62	20		6.49***
L middle temporal gyrus	−58	−2	−22	1111	7.88***
L medial temporal pole	−48	16	−30		7.37***
L middle temporal gyrus	−62	−8	−14		6.98***
R medial temporal pole	40	20	−34	477	5.49**
R medial temporal pole	48	16	−30		5.43**
R middle temporal gyrus	56	−6	−20		5.35**
L mid orbital gyrus, including vmPFC	0	60	−4	898	5.96***
L rectal gyrus, including mPFC	0	40	−16		5.88***
L mid orbital gyrus, including vmPFC	-2	52	−6		5.23**
Solo Sequencing < Nonsocial Sequencing					
L middle temporal gyrus	-56	−60	2	805	6.80***
L inferior temporal gyrus	-48	−44	−14		5.09*
L supramarginal Gyrus	-60	−38	36	903	6.84***
L supramarginal Gyrus	−62	−26	32		6.15***
L inferior parietal lobule	−52	−44	38		5.72**
L IFG p. triangularis	−46	36	18	521	6.22***
L IFG p. triangularis	−50	38	8		5.63**
Interactive Selection-only > Nonsocial Selection-only					
L precuneus	−8	−50	16	815	5.44*
Interactive Selection-only < Nonsocial Selection-only					
---					
Solo Selection-only > Nonsocial Selection-only					
---					
Solo Selection-only < Nonsocial Selection-only					
---					
Main effects: Social > Nonsocial					
ROI: cerebellum IX	4	−58	−46	36	4.36*
ROI: cerebellum IX	−2	−56	−44		3.91
L precuneus	−4	−50	18	1294	6.63***
L PCC	−2	−48	28		5.51**
L precuneus	−4	−48	10		5.34**
L middle temporal gyrus	−60	−10	-14	1116	7.10***
L medial temporal pole	−46	16	−32		6.35***
L middle temporal gyrus	−58	−2	−20		6.20***
L mid orbital gyrus, including vmPFC	−4	60	−4	346	5.34**
Main effects: Social < Nonsocial					
L IFG p. triangularis	−48	34	12	229	5.57**
L IFG p. triangularis	−44	30	18		5.17*

Note: Coordinates refer to the MNI (Montreal Neurological Institute) stereotaxic space reported for all (sub)clusters in cerebellar and mentalizing areas, and only for peak clusters for other areas. Whole-brain analysis thresholded at cluster-defining uncorrected threshold of *p* < 0.001, followed by a cluster-wise FWE corrected *p* < 0.05 and voxel extent ≥10. L = left, R = right, PCC = posterior cingulate cortex, TPJ = temporoparietal junction, mPFC = medial prefrontal cortex, dmPFC = dorsal medial prefrontal cortex, vmPFC = ventral medial prefrontal cortex, MCC = mid-cingulate cortex, IFG = inferior frontal gyrus. *p < 0.05; **p < 0.01; ***p < 0.001 (peak FWE corrected).

#### Solo Sequencing versus Nonsocial Sequencing

As predicted, the whole brain analysis for the Solo Sequencing > Nonsocial Sequencing contrast (Fig. 3C; Table 3) revealed activations in cerebellar mentalizing areas in lobule IX, and in cerebral mentalizing areas in the precuneus, PCC, vmPFC, and mPFC. We found further activations in the bilateral aMTG and right medial temporal pole. The opposite contrast, Solo Sequencing < Nonsocial Sequencing, revealed activations in the left pMTG, left supramarginal gyrus, and left IFG pars Triangularis.

#### Interactive Selection-only versus Nonsocial Selection-only

The whole brain analysis for the Interactive Selection-only > Nonsocial Selection-only contrast (Table 3) revealed activations in the cerebral mentalizing areas in the precuneus and, as predicted, no activation in cerebellar areas. ROI analysis revealed no further activations. The opposite contrast Interactive Selection-only < Nonsocial Selection-only, revealed no activations for whole brain and ROI analysis.

#### Solo Selection-only versus Nonsocial Selection-only

Both the Solo Selection-only > Nonsocial selection-only (Table 3) and the opposite Solo Selection-only < Nonsocial selection-only contrast revealed no activations in either whole brain or ROI analysis.

#### Main effects: Social versus Nonsocial

To evaluate whether cerebral mentalizing areas are involved in social conditions irrespective of sequencing, we compare all Social conditions (Interactive Sequencing, Interactive Selection-only, Solo Sequencing, Solo Selection only) > Nonsocial conditions (Nonsocial Sequencing, Nonsocial Selection-only; Fig. 3D; Table 3). The whole-brain analysis revealed, as predicted, activations in cerebral mentalizing area activations in the precuneus, PCC and vmPFC. Unexpectedly, the ROI analysis further revealed activations in cerebellar lobule IX. We found further activations in the aMTG. The opposite contrast, Social contrast < Nonsocial, revealed activations in the left IFG pars triangularis.

### Novel hypothesis: Interactive versus Solo contrasts

Our novel hypothesis was that the cerebellum, along with some mentalizing areas (e.g., TPJ, dmPFC) might be more involved when processing behavioral descriptions about preferences of two people (Interactive condition) than one person (Solo conditions). Note that as before, for the cerebellum, we expect these contrasts only during sequencing, whereas for the cerebrum, we expect this irrespective of sequencing.

#### Interactive Sequencing versus Solo Sequencing

As predicted, the whole brain analysis of the Interactive Sequencing > Solo Sequencing contrast (Fig. 3A; Table 4) revealed activations in cerebellar mentalizing areas in the bilateral cerebellar Crus 1, left Crus 2, and lobule IX, and in cerebral mentalizing areas in the left TPJ and precuneus. ROI analysis further revealed activations in the mPFC and dmPFC. We also found activations in the bilateral aMTG. The reverse contrast, Interactive Sequencing > Solo Sequencing, revealed no activations in either the whole brain or ROI analysis.

**Table 4:** Whole brain and ROI analyses Interactive versus Solo conditions

Brain label	MNI coordinates			Voxels	Max t
	x	y	z		
Interactive Sequencing > Solo Sequencing					
L cerebellum Crus 2	-16	-88	-34	354	5.46**
L cerebellum Crus 1	-24	-80	-32		5.42**
L cerebellum Crus 2	-36	-80	-34		5.22**
R cerebellum Crus 1	28	-82	-30	213	5.22**
ROI: L cerebellum IX	-6	-56	-42	25	5.16**
ROI: R cerebellum IX	6	-52	-42	7	3.7
L angular gyrus, including TPJ	-38	-58	24	453	6.55***
L precuneus	0	-52	40	1188	6.50***
L precuneus	-8	-58	36		6.41***
L MCC	-10	-48	32		5.57**
L middle temporal gyrus	-60	-10	-12	572	6.03***
R middle temporal gyrus	60	-4	-24	805	5.95***
R middle temporal gyrus	54	-14	-14		5.20**
ROI: dmPFC	-6	50	42	202	4.52*
ROI: mPFC	6	52	22	62	4.3
Interactive Sequencing < Solo Sequencing					
---					
Interactive Selection-only > Solo Selection-only					
---					
Interactive Selection-only < Solo Selection-only					
---					
Main effects: Interactive > Solo					
ROI: precuneus	-6	-62	36	63	3.78
Main effects: Interactive < Solo					
---					

Coordinates refer to the MNI (Montreal Neurological Institute) stereotaxic space reported for all (sub)clusters in cerebellar and mentalizing areas, and only for peak clusters for other areas. Whole-brain analysis thresholded at cluster-defining uncorrected threshold of *p* < 0.001, followed by a cluster-wise FWE corrected at *p* < 0.05 and voxel extent ≥ 10. L = left, R = right, TPJ = temporoparietal junction, mPFC = medial prefrontal cortex, dmPFC = dorsomedial prefrontal cortex, MCC = mid-cingulate cortex. *p < 0.05; **p < 0.01; ***p < 0.001 (peak FWE corrected)

#### Interactive Selection-only versus Solo Selection-only

For both the Interactive Selection-only > Solo Selection-only (Table 4) and the opposite Interactive Selection-only < Solo Selection-only contrast, we found no activations for either whole brain or ROI analysis.

#### Main effects: Interactive versus Solo

To evaluate whether cerebral mentalizing areas are more involved in Interactive than Solo conditions irrespective of sequencing, we compared all Interactive (i.e., Interactive Sequencing, Interactive Selection-only) > Solo (Solo Sequencing, Solo Selection-only) conditions (Fig. 3C; Table 4). The whole brain analysis revealed no activations. The ROI analysis, however, revealed activations in the precuneus. The opposite Interactive < Solo contrast revealed no activations for either whole brain or ROI analysis.

### Sequencing versus Selection-only contrasts

We hypothesized that the posterior cerebellar Crus, especially Crus 1 would be robustly activated during mentalizing when participants generated a sequence of possible future preference-related behaviors (Sequencing) rather than when they merely selected one out of two preference-related options (Selection-only). Contrary to the hypothesis, none of the predicted effects were significant.

#### Interactive Sequencing versus Interactive Selection-only

The Interactive Sequencing > Interactive Selection-only contrast (Fig. 3D; Table 5) revealed no activations for either whole brain or ROI analysis. The opposite contrast, Sequencing < Interactive Selection-only, revealed activations in the superior occipital gyrus.

**Table 5:** Whole brain and ROI analyses comparing sequencing versus selection-only conditions

Brain label	MNI coordinates			Voxels	Max t
	x	y	z		
Interactive Sequencing > Interactive Selection-only					
---					
Interactive Sequencing < Interactive Selection-only					
L superior occipital gyrus	-10	-96	10	817	5.21
Solo Sequencing > Solo Selection-only					
---					
Solo Sequencing < Solo Selection-only					
Nonsocial Sequencing > Nonsocial Selection-only					
R Precuneus	8	-64	48	355	5.35*
Nonsocial Sequencing < Nonsocial Selection-only					
L superior occipital gyrus	-10	-98	10	1652	8.72***
R cuneus	10	-96	16		7.49***
L middle occipital gyrus	-22	-94	12		5.71*
R MCC	6	16	36	679	5.64**
R superior medial gyrus, including dmPFC	4	62	28	213	5.35*
Main effects: Sequencing > Selection-only					
---					
Main effects: Sequencing < Selection-only					
L cuneus	-8	-96	12	2326	8.72***
R cuneus	10	-96	16		7.49***
L calcarine gyrus	4	-86	2		5.71*
L hippocampus	-36	-26	-2	139	5.35
R IFG pars opercularis	38	14	10	1293	5.644*

Coordinates refer to the MNI (Montreal Neurological Institute) stereotaxic space reported for all (sub)clusters in cerebellar and mentalizing areas, and only for peak clusters for other areas. Whole-brain analysis thresholded at cluster-defining uncorrected threshold of *p* < 0.001, followed by a cluster-wise FWE corrected at *p* < 0.05 and voxel extent ≥10. L = left, R = right, MCC = mid-cingulate cortex, IFG = inferior frontal gyrus. *p < 0.05; **p < 0.01; ***p < 0.001 (peak FWE corrected)

#### Solo Sequencing versus Solo Selection-only

Both the Solo Sequencing > Solo Selection-only (Fig. 3E; Table 5) and the opposite Solo Sequencing < Solo Selection-only contrast revealed no activations for either the whole brain or the ROI analysis.

#### Main effects: Sequencing versus Selection-only

In the Sequencing (Interactive Sequencing, Solo Sequencing, Nonsocial Sequencing) > Selection-only (Interactive Selection-only, Solo Selection-only, Nonsocial Selection-only) contrast (Fig. 3F; Table 5), we found no activations in either whole brain or ROI analysis. The opposite contrast, Sequencing < Selection-only revealed activations in the cuneus, calcarine gyrus, left hippocampus, and right IFG pars opercularis.

## Discussion

This study investigated the neural correlates involved in predicting social action sequences based on known preferences of interacting or individual persons. In line with previous research and our general hypothesis, the results confirm that cerebellar (Crus, lobule IX) and cerebral (mPFC, TPJ, precuneus) mentalizing areas were involved in predicting social actions. More importantly, consistent with our novel hypothesis, this was especially the case when predicting the interactions of two persons rather than actions of a single person based on their preferences. Although the direct comparison between sequencing versus nonsequencing predictions in a social context did not reach significance, it is important to note that the cerebellar mentalizing activations were only found during predictions of interactive and individual (vs. nonsocial) action sequences, and not in the parallel non-sequencing (i.e., selection-only) contrasts, consistent with our general hypothesis.

### Predicting social actions based on preferences activates the Mentalizing network

Our results confirm previous cerebellar research that posterior and inferior posterior cerebellar of the mentalizing network have a domain-specific function in predicting sequences of social behavior, specifically in social mentalizing (Van Overwalle et al., 2014; Van Overwalle, Baetens, et al., 2015a). This social specificity is further supported by the coactivation of cerebral social mentalizing areas, irrespective of sequencing.

#### Stronger mentalizing cerebellar activation in interactive than solo sequence prediction

In line with our novel hypothesis, we observed strong cerebellar Crus 1 and 2 (and lobule IX) activation during interactive sequencing prediction (compared with selection-only prediction), and more so than during the prediction of an individual person’s solitary actions. The activation of cerebellar Crus 1 and 2 (and lobule IX in the interactive sequencing condition is in line with our prior prediction research based on a person’s traits and intentions (Haihambo et al., 2022, 2023). However, surprisingly, in the individual condition lobule IX activation, but not Crus activation was found when compared against a non-social sequence condition. This suggests that cerebellar Crus mentalizing areas may be selectively activated when predicting social interactions rather than individual actions. While the role of the Crus area is well established in social mentalizing as playing a role in identifying and reproducing social actions in the correct chronological order (for an overview, see Van Overwalle et al., 2021), the lack of significant Crus activation in the individual condition might be due to the fact that prediction is less critical in solitary conditions, as mishaps can be easily corrected. In addition to these neurological findings, we also found behavioral differences: participants were more accurate in the Solo conditions, followed by the Interactive conditions. This suggests that predicting interactive social actions may be more challenging than predicting solo actions. This further reflects the additional complexity involved in predicting interactive events requiring the tracking of multiple mental states resulting in increased activations in cerebellar mentalizing areas and the dmPFC (Li et al., 2014). Of note, our results undermine a cerebellar role of purely linguistic sequence processing in the present experiment, as this cannot explain the significant differences between interactive and solo conditions that all consisted of similar sets of sentences.

When comparing the individual condition (and also in the interactive conditions) to its non-social counterpart, we only found robust activation of the cerebellar lobule IX. The function of lobule IX is not well understood. Although activation in this area clearly overlaps with the default mode network (Buckner et al., 2011; Habas et al., 2009; Stephen et al., 2018 see Fig. 3), it is not immediately clear what specific role lobule IX plays in mentalizing. This area is seldom activated in identifying sequences during trait inferences (Pu et al., 2020; Pu, Heleven, Ma, et al., 2022a; Pu, Ma, Heleven, et al., 2022c), belief inferences (Heleven et al., 2019; Ma, Pu, Haihambo et al., 2021), or goal-directed behavior (Li et al., 2021, 2022). In contrast, we found similar lobule IX activation in our previous studies on predictive mentalizing based on traits (Haihambo et al., 2022) and intentions (Haihambo et al., 2023). Additionally, cerebellar lobule IX activation was also found in other domains such as autobiographical memory when participants had to imagine the future (Addis et al., 2007). Indeed, functional parcellations, such as those by Ji et al. (2019) and Buckner et al. (2011), associate lobule IX with mentalizing (specifically in clusters activated in the present study). Taking these results together, we suggest that lobule IX is involved in mentalizing during self and other referential thinking requiring future-oriented thought. The role of lobule IX also could be construed as contributing to language processing, as demonstrated by several sources (Diedrichsen & Zotow, 2015; King et al., 2019). However, on closer inspection, these studies use stories describing social scenarios, which makes their characterization as purely language very limited. To better understand the role of cerebellar lobule IX in mentalizing and explicit prediction, and to distinguish it from purely language processes, further studies should disentangle how social task demands may modulate lobule IX activation, for instance, to disentangle the contribution of this area during self or other-directed prediction versus future-directed thought in general.

#### Mentalizing cerebrum in social prediction irrespective of sequencing

While activation of cerebellar mentalizing areas was mainly confined to predicting social sequences in our prediction task, activation of cerebral mentalizing areas was observed during social mentalizing irrespective of sequences or not, in line with our general hypothesis derived from prior work. When comparing social versus nonsocial conditions, we found activation in cerebral mentalizing area including the precuneus, temporal pole, and vmPFC. The precuneus has consistently been activated in studies that involved mental imagery of scenes that set the context for social action (Molenberghs et al., 2016; Schurz et al., 2014; Van Overwalle & Baetens, 2009). The temporal pole has been associated with representations of specific knowledge, such as norms and values, about others and the world (Monticelli et al., 2021; Schurz et al., 2014). The vmPFC is related to thinking about similar others, and in processing our own preferences (Heleven & Van Overwalle, 2019; Ito et al., 2022; Mitchell et al., 2006; Van Overwalle, 2009) All these processes are involved in making predictions about other persons’ actions based on their preferences, such as imaging an adequate scene, knowing the boundaries set by social norms and values, and thinking about a persons’ inner preferences.

When interactive sequences are involved, we found additional activations in the dmPFC, TPJ, and precuneus, which were not observed in the individual solo context, consistent with our novel hypothesis. We anticipated that the dmPFC would be selectively activated for social interactions as attention is centered on other persons about whom we mentalize (Li et al., 2014). Activation of the dmPFC also has been observed in other studies that compared observations of solo versus interactive social scenes (Iacoboni et al., 2004) and preference related tasks (Izuma & Adolphs, 2013; Kang et al., 2013). For example, a study by Kang et al. (2013) found that the dmPFC was robustly activated when participants guessed an unfamiliar person’s preferences for an item (e.g., books). Moreover, there is some evidence to suggest that the mPFC is sensitive to sequences during retrieval (Reeders et al., 2021). As anticipated, we also observed that the TPJ was preferentially activated during social interactions. This is not surprising, since earlier research found that the TPJ is more activated when two, rather than one person, have divergent false beliefs (Özdem et al., 2019). In addition, we also find activation in the precuneus, which is involved in mentally representing social scenes (Molenberghs et al., 2016; Schurz et al., 2014; Van Overwalle & Baetens, 2009). Previous studies also found that the precuneus was activated when participants observed others in joint actions (Petrini et al., 2014), suggesting that the precuneus plays a key role in generating and maintaining a mental representation of the social context in interactions (Schurz & Perner, 2015).

#### Stronger mentalizing cerebellar activation for predicting sequences?

A number of researchers have suggested that the main, and perhaps most basic function of the cerebellum is in sequential processing across a number of functional domains, such as in motor perception and execution, cognitive control, and mentalizing (Clausi et al., 2019; Molinari et al., 2008; Van Overwalle et al., 2021). In line with this, we investigated the sequencing function of the cerebellum, hypothesizing that the cerebellar Crus area would be activated when participants had to generate the sequence of preference-directed (inter)actions.

To our surprise however, and contrary to the sequencing hypothesis, our neuroimaging results did not reveal cerebellar activations for either whole brain or ROI analysis when comparing Sequencing conditions to their nonsequencing Selection-only counterpart. However, we did observe the hypothesized cerebellar recruitment in each of the interactive and solo versus nonsocial contrasts only when sequences were involved (e.g., Interactive/Solo Sequencing vs. Nonsocial Sequencing), and not when sequencing was not required (e.g., Interactive/Solo Selection-only vs. Nonsocial Selection-only). These findings suggest that the cerebellum's involvement in social mentalizing is contingent upon the presence of specific sequencing demands imposed by the task. Future studies should further investigate the sequencing-specific role of the cerebellum during social mentalizing and the boundaries of this function.

Behaviorally, we find that participants are faster, but less accurate in Selection-only tasks compared to Sequencing tasks. We suggest that this is likely due to a speed-accuracy tradeoff effect (Zimmerman, 2011). Specifically, in the context of the relatively easier selection-only task, where the generation of a sequence is not required and thus demands comparatively less cognitive effort, participants may prioritize speed over accuracy. Consequently, they may be less attentive to subtle nuances in the presented information, leading to a greater likelihood of making careless mistakes.

#### Limitations and questions

While the present study provides novel insights into the role of cerebellar and cerebral mentalizing areas in predicting preference related behaviors that involve either a single protagonists engaging in behaviors alone or two protagonists interacting, several limitations need to be mentioned. First, our material often included the same preference wording (e.g., hiking) in the prompt sentence and behavioral sentences (e.g., So, Ytol looked for a hiking trail nearby), unlike our previous prediction tasks that avoided the same words in the prompt and behavioral sentences in order to induce strong mentalizing inference processes (Haihambo et al., 2022, 2023). However, it is almost impossible to eliminate specific terms related to the preferred object or activity. This could have made sequencing redundant, as participants merely had to select the sentence that included “hiking.” This could have made the task requirements for the Sequencing and (nonsequencing) Selection-only tasks too similar for distinct cerebellar activation to be observed. Second, ten trials per conditions may be quite limited, resulting in insufficient power to drive cerebellar activation. Although this is an important consideration, it is also worth noting that a number of studies discussed above had comparable number of trials and participants. For example, in two studies also focusing on social action prediction, Haihambo et al. (2022, 2023) included 27 and 26 participants respectively, with 11 trials per condition, and they found significant differences between Sequencing and Selection-only. Similarly, a preference related task by Jenkins and Mitchell (2010) included 15 participants and 15 trials. So, while having more trials and participants may increase the power of the study and provide more robust results, other studies with similar trials have been able to provide meaningful insights into social action prediction with and without preference related information.

## Conclusions

We investigated cerebellar and cerebral mentalizing areas in predicting preferences-based social interactions. Our findings supported the role of the posterior cerebellar Crus 1, Crus 2, and lobule IX, and the dmPFC in social processes. For the first time, we demonstrated that this role was stronger in person-to-person interactions than activities of a single person. We were, however, not able to support the sequencing hypothesis of the cerebellum in this study. Our results also point to specialized roles within the mentalizing areas of the cerebellum, in particular, the role of cerebellar lobule IX in predicting future social actions regardless of sequencing requirements and further advance our understanding of the mentalizing and sequencing role of the cerebellum during social action prediction.

## Data Availability

All (pseudonymized or anonymous) data are available upon request, excluding data that allow identifying individual participants. If relevant, all manuals and code for processing the data also are available.
